# High Reliability and Breakdown Voltage of GaN HEMTs on Free-Standing GaN Substrates

**DOI:** 10.3390/nano15241882

**Published:** 2025-12-15

**Authors:** Shiming Li, Mei Wu, Ling Yang, Hao Lu, Bin Hou, Meng Zhang, Xiaohua Ma, Yue Hao

**Affiliations:** National Engineering Research Center of Wide Band-Gap Semiconductor, Faculty of Integrated Circuit, Xidian University, Xi’an 710126, China; smli@stu.xidian.edu.cn (S.L.);

**Keywords:** gallium nitride (GaN), high electron mobility transistors (HEMTs), free-standing GaN substrate, breakdown voltage (BV), dislocation, reliability

## Abstract

Gallium nitride (GaN)-based high electron mobility transistors (HEMTs) are pivotal for next-generation power-switching applications, but their reliability under high electric fields remains constrained by lattice mismatches and high dislocation densities in heterogeneous substrates. Herein, we systematically investigate the electrical performance and reliability of GaN-on-GaN HEMTs in comparison to conventional GaN-on-SiC HEMTs via DC characterization, reverse gate step stress, off-state drain step stress, and on-state electrical stress tests. Notably, the homogeneous epitaxial structure of GaN-on-GaN devices reduces dislocation density by 83.3% and minimizes initial tensile stress, which is obtained through HRXRD and Raman spectroscopy. The GaN-on-GaN HEMTs exhibit a record BFOM of 950 MW/cm^2^, enabled by a low specific on-resistance (*R*_ON-SP_) of 0.6 mΩ·cm^2^ and a high breakdown voltage (BV) of 755 V. They withstand gate voltages up to −200 V and drain voltages beyond 200 V without significant degradation, whereas GaN-on-SiC HEMTs fail at −95 V (reverse gate stress) and 150 V (off-state drain stress). The reduced dislocation density suppresses leakage channels and defect-induced degradation, as confirmed by post-stress Schottky/transfer characteristics and Frenkel–Poole emission analysis. These findings establish GaN-on-GaN technology as a transformative solution for power electronics, offering a unique combination of high efficiency and long-term stability for demanding high-voltage applications.

## 1. Introduction

Gallium nitride (GaN)-based high electron mobility transistors (HEMTs) have emerged as cornerstone devices for next-generation microwave and power switching systems, leveraging their inherent advantages of a high electron saturation velocity (~2 × 10^7^ cm/s), dense two-dimensional electron gas (2DEG, ~1 × 10^13^ cm^−2^), and a robust critical electric field (~3.3 MV/cm)—properties that far outperform traditional silicon (Si) and silicon carbide (SiC) power devices [1,2,3]. With the rapid development of electric vehicles (EVs), renewable energy inverters, and industrial power converters, there is an urgent demand for power semiconductors that combine high breakdown voltage (BV), low on-resistance, and long-term reliability under extreme operating conditions—requirements that GaN HEMTs are uniquely positioned to meet, yet their full potential remains constrained by material and structural limitations [4,5].

For decades, GaN HEMTs have been predominantly fabricated on heterogeneous substrates (e.g., 4H-SiC or Si) due to the high cost and scalability challenges of free-standing GaN (FS-GaN) substrates [6,7,8]. However, lattice mismatches (e.g., ~3.5% between GaN and 4H-SiC) and thermal expansion coefficient differences (e.g., ~50% mismatch between GaN and Si) between the AlGaN/GaN epitaxial stack and heterogeneous substrates inevitably induce high-density crystal defects, including threading dislocations and stacking faults [9]. These defects elevate the dislocation density to ~10^8^–10^9^ cm^−2^, which not only increases specific on-resistance (*R*_ON-SP_) and off-state leakage currents, but also induces a severe current collapse under dynamic operation—issues that lead to increased power loss, thermal runaway, and even premature device failure in high-voltage systems, limiting the adoption of GaN HEMTs in mission-critical applications [10,11].

To address these challenges, FS-GaN substrates have gained traction in recent years, enabling homoepitaxial growth of AlGaN/GaN stacks that minimize lattice mismatch and reduce the dislocation density [12,13,14,15]. Our prior work introduced a Fe/C co-doped GaN buffer layer design that effectively mitigates interfacial charge accumulation and suppresses buffer leakage in GaN-on-GaN HEMTs, demonstrating their potential for radio frequency (RF) and power-switching applications [16]. However, the literature on GaN-on-GaN HEMTs remains limited in two key aspects: first, most studies focus solely on static DC performance (e.g., BV and *R*_ON-SP_) and lack systematic evaluation of reliability under multi-stress conditions (reverse gate bias, off-state drain stress, and on-state power dissipation) that devices encounter in real-world operation; second, the direct correlation between dislocation density (a critical material quality metric) and stress-induced degradation mechanisms has not been fully established, leaving a gap in understanding how to optimize epitaxial structures for enhanced robustness.

In this study, we aim to fill these gaps by systematically investigating the electrical performance and reliability of GaN-on-GaN HEMTs in comparison to conventional GaN-on-SiC devices. We quantify dislocation density via high-resolution X-ray diffraction (HRXRD) and link it to device behavior under diverse stress tests, while also characterizing epitaxial strain via Raman spectroscopy to elucidate the role of initial structural stress in degradation. By focusing on both material-level properties and device-level reliability, this work not only validates the superiority of GaN-on-GaN technology but also provides a roadmap for optimizing GaN HEMTs for high-voltage, high-reliability power electronics applications.

## 2. Experimental Details

### 2.1. Device Structure and Fabrication Process

**Figure 1:**
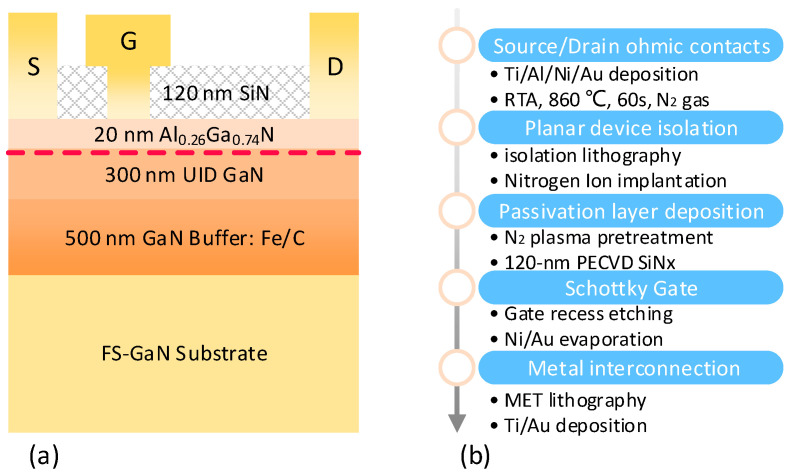
(**a**) The schematic cross-sectional structure of the GaN-on-GaN HEMTs. (**b**) Process flow for the fabricated GaN HEMTs in this work.

Figure 1a presents the cross-sectional structure of GaN HEMTs grown on a two-inch FS-GaN substrate. The epitaxial stack consists of a 20 nm Al_0.26_Ga_0.74_N barrier layer, a 300 nm unintentionally doped (UID) GaN channel layer, a 500 nm Fe/C co-doped GaN buffer layer, and a 350 μm thick FS-GaN substrate. For comparative analysis, the same epitaxial structure was grown on a three-inch SiC substrate (GaN-on-SiC) via Metal Organic Chemical Vapor Deposition (MOCVD), with a 60 nm AlN nucleation layer inserted to improve epitaxial quality. Hall effect measurements prior to device fabrication yielded carrier concentrations of 1.1 × 10^13^ cm^−2^ (GaN-on-SiC) and 1.0 × 10^13^ cm^−2^ (GaN-on-GaN), along with electron mobilities of 1570 cm^2^/V·s and 1680 cm^2^/V·s, respectively.

Both device types were fabricated by using an identical process flow to isolate substrate-induced effects. As illustrated in Figure 1b, the fabrication sequence began with the deposition of Ti/Al/Ni/Au layers for source/drain ohmic contacts, followed by rapid thermal annealing (RTA) at 860 °C in a N_2_ atmosphere for 60 s. Planar isolation was achieved via multi-energy nitrogen ion implantation after lithography. The wafer surface was pretreated with N_2_ plasma before depositing a 120 nm SiN_x_ passivation layer, using plasma-enhanced chemical vapor deposition (PECVD). The gate foot defined by lithography was opened using CF_4_-based plasma etching, followed by a T-shaped Ni/Au gate geometry for the Schottky contact. Ti/Au metal interconnections (MET) were formed as the final step. The standard device dimensions were as follows: gate-source spacing (L_gs_) = 1.25 μm, gate length (L_g_) = 0.5 μm, and source-drain spacing (L_sd_) = 8 μm. For reliability tests, devices with uniform dimensions (L_gs_ = L_gd_ = L_g_ = 1 μm) were used to ensure consistent stress conditions. Based on the current experimental data, the growth process of GaN-on-GaN structures (using MOCVD) exhibits excellent compatibility with the growth processes of GaN structure on SiC substrates. Furthermore, the actual fabrication processes for both devices are performed on the same production line, demonstrating process compatibility. This proved that the MOCVD-based growth/fabrication process offers favorable affordability.

### 2.2. Epitaxial Material Characterization

**Figure 2:**
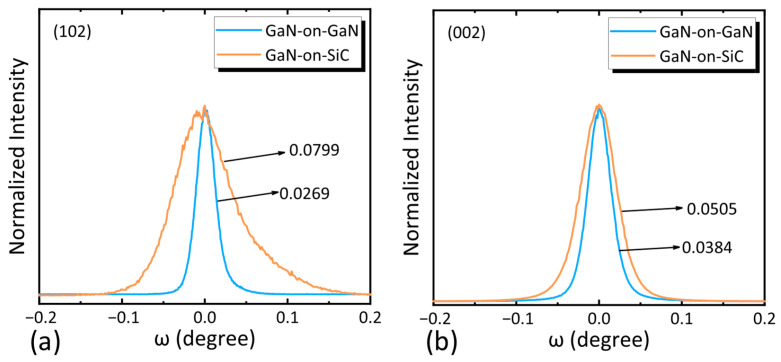
Rocking curves of the (**a**) (102) plane and (**b**) (002) plane in the GaN-on-GaN structure and the GaN-on-SiC structure.

HRXRD was employed to evaluate the crystalline quality of the AlGaN/GaN epitaxial layers, with full-width at half-maximum (FWHM) values of the (002) and (102) planes measured to calculate dislocation densities [12]. The total dislocation density (*D*_dis_), screw dislocation density (*D*_screw_), and edge dislocation density (*D*_edge_) were derived using the following equations [17]:

(1) $$D_{\mathrm{screw}}\;=\;\frac{\beta_{\left(002\right)}^{2}}{9b_{\mathrm{screw}}^{2}},D_{\mathrm{edge}}=\frac{\beta_{\left(102\right)}^{2}}{9b_{\mathrm{edge}}^{2}}$$

(2) $$D_{\mathrm{dis}}=D_{\mathrm{screw}}+D_{\mathrm{edge}}$$

where *b*_screw_ = 0.52 nm and *b*_edge_ = 0.32 nm are the Burgers vector lengths and *β* denotes the FWHM values from the XRD rocking curves. The rocking curves for the two samples are shown in Figure 2. The GaN-on-SiC structure exhibited FWHM values of 182 arcsec (002) and 288 arcsec (102), corresponding to a total dislocation density of 2.35 × 10^8^ cm^−2^. In contrast, the GaN-on-GaN structure showed narrower FWHM values (125 arcsec for (002) and 97 arcsec for (102)), resulting in a significantly lower total dislocation density of 3.93 × 10^7^ cm^−2^—an 83.3% reduction compared to GaN-on-SiC structure. This confirms the superior lattice matching and crystalline quality of homoepitaxial GaN-on-GaN structures.

**Figure 3:**
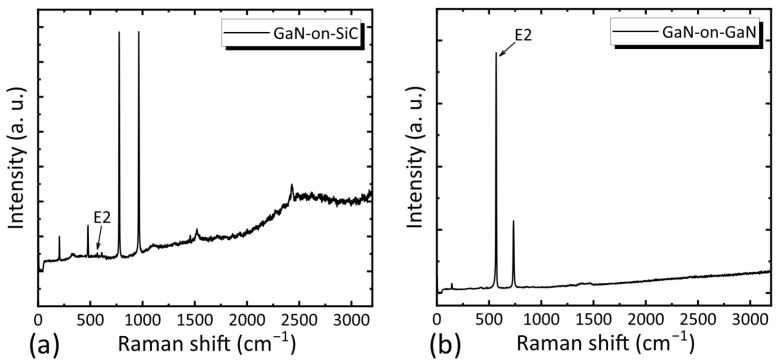
Raman spectrum of the (**a**) GaN-on-SiC structure and the (**b**) GaN-on-GaN structure.

**Figure 4:**
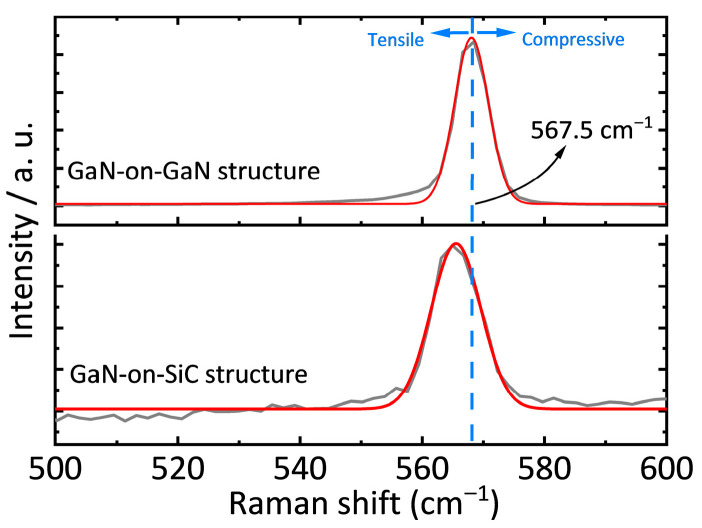
Raman shifts in GaN film in the GaN-on-GaN structure and the GaN-on-SiC structure.

Raman spectroscopy was used to characterize the strain state of the AlGaN barrier and GaN buffer layers, with the E_2_ (TO) mode employed as a strain marker. The stress (σ) was calculated using the following [18]:

(3) $$\sigma \;=\;\frac{\Delta \omega }{k}$$

where Δω = ω_measured_ − ω_stress-free_, ω_stress-free_ = 567.5 cm^−1^ [19] and *k* = −3.4 cm^−1^GPa^−1^ (stress coefficient for GaN [20]). The full Raman spectra of the two samples are shown in Figure 3. To highlight the details of the E_2_ mode Raman peak, the Raman spectrum near 567.5 cm^−1^ has been magnified, as illustrated in Figure 4. The GaN-on-GaN structure exhibited a Raman peak at 567.6 cm^−1^, corresponding to a near-stress-free state (−0.03 GPa). In contrast, the GaN-on-SiC structure showed a peak shift to 565.5 cm^−1^, indicating significant tensile stress (0.59 GPa) arising from thermal mismatch and lattice mismatch. The AlGaN layer in the GaN-on-GaN structure experienced only lattice mismatch-induced tensile stress, whereas the GaN-on-SiC structure accumulated additional residual tensile stress from the GaN layer—further highlighting the structural advantage of FS-GaN substrates.

## 3. Results and Discussion

### 3.1. DC Characterization

**Figure 5:**
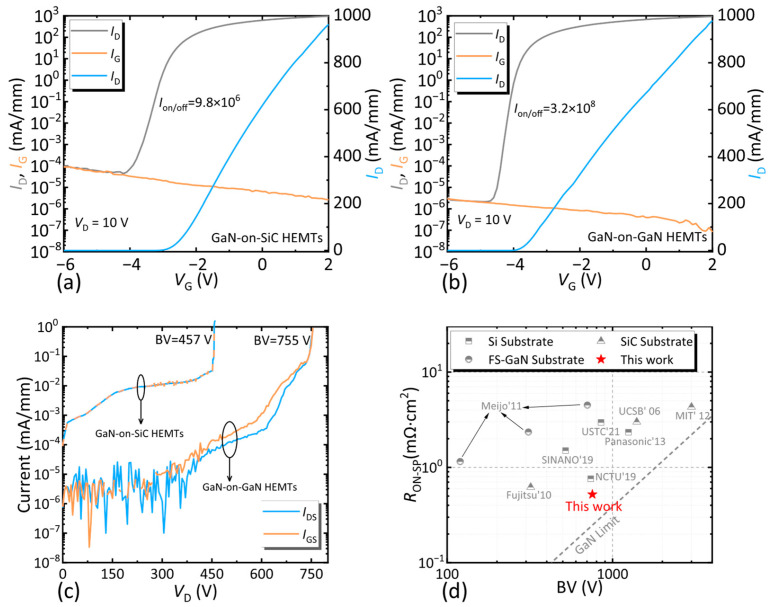
The linear and semi-log scale of *I*_D_–*V*_G_ transfer performance of (**a**) the GaN-on-SiC HEMTs and (**b**) the GaN-on-GaN HEMTs. (**c**) BV of GaN HEMTs on different substrates with *L*_sd_ = 8 μm. (**d**) Comparison of *R*_ON-SP_ versus BV from different research groups.

DC electrical properties were measured using Keithley B1500A and B1505A semiconductor device analyzers (Keithley Instruments (now part of Tektronix, Inc.), Solon, OH, USA). Figure 5a,b show the linear and semi-logarithmic transfer characteristics of the GaN-on-SiC and GaN-on-GaN HEMTs, respectively, measured at a drain bias (*V*_D_) of 10 V. For the GaN-on-SiC HEMTs, the off-state drain leakage current (*I*_D, OFF_) at *V*_G_ = −6 V was 9.8 × 10^−5^ mA/mm, the maximum saturated drain current density (*I*_D, max_) at *V*_G_ = 2 V was 960 mA/mm, and the on/off current ratio (*I*_on/off_) was 9.8 × 10^6^. The GaN-on-GaN HEMTs exhibited a much lower *I*_D, OFF_ (2.6 × 10^−6^ mA/mm), a comparable *I*_D, max_ (979 mA/mm), and a significantly higher *I*_on/off_ (3.2 × 10^8^). This improvement in off-state performance is attributed to reduced dislocation density—dislocations act as conductive channels in GaN HEMTs, and their minimization effectively suppresses leakage currents [21]. The breakdown voltage (BV) was extracted under a leakage current criterion of 1 mA/mm (Figure 5c). GaN-on-GaN HEMTs with L_sd_ = 8 μm achieved a BV of 755 V, whereas GaN-on-SiC HEMTs of the same dimensions reached a maximum BV of 457 V. The superior breakdown performance stems from the lattice-matched homoepitaxial structure, which eliminates defect-induced leakage paths. Combined with the low *R*_ON-SP_ of 0.6 mΩ·cm^2^, the GaN-on-GaN HEMTs achieved a BFOM of 950 MW/cm^2^ (calculated as BFOM = BV ^2^/*R*_ON-SP_)—the highest value reported to date for GaN-on-GaN HEMTs (Figure 5d) [22,23,24,25,26,27], underscoring their potential for high-efficiency power switching.

### 3.2. Reverse Gate Step Stress Characterization

Firstly, reverse gate step stress tests were performed to evaluate the device’s reliability under high electric fields, with a focus on degradation induced by the reverse piezoelectric effect [28]. During testing, *V*_D_ = vs. = 0 V, and the gate voltage (*V*_G_) was stepped from 0 V to −200 V in increments of −5 V every 5 min. Gate current (*I*_G_), source current (*I*_S_), and drain current (*I*_D_) were monitored continuously, with a critical voltage (*V*_critical_) defined as being the point where current noise was detected [28].

**Figure 6:**
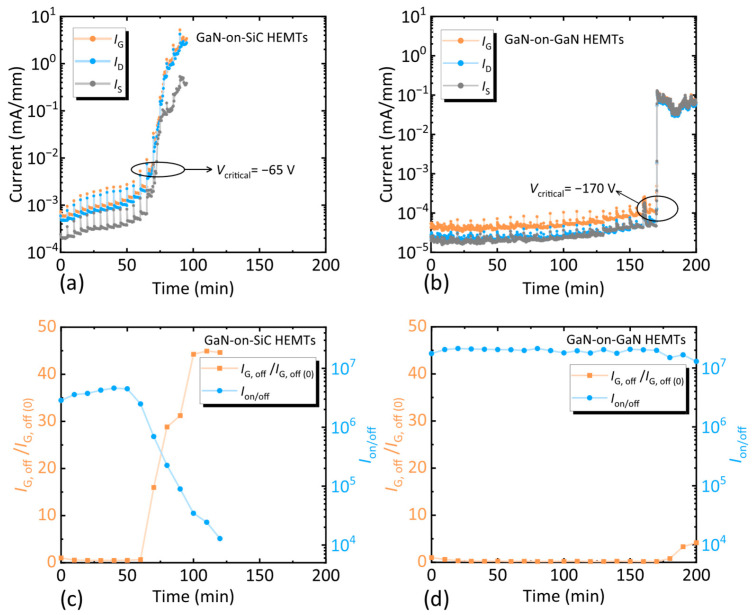
Changes in *I*_G_, *I*_S_, and *I*_D_ of (**a**) the GaN-on-SiC HEMTs and (**b**) the GaN-on-GaN HEMTs in reverse gate step stress experiments. Relationship between the *I*_on/off_ and normalized *I*_G, off_ of (**c**) the GaN-on-SiC HEMTs and (**d**) the GaN-on-GaN HEMTs as a function of reverse gate step stress, respectively. *I*_G, off_/*I*_G, off (0)_ is defined as the ratio of the *I*_G, off_ after each step post-stress to the stress-free *I*_G, off_.

As shown in Figure 6a, GaN-on-SiC HEMTs failed at *V*_G_ = −95 V, with current noise observed as early as *V*_G_ = −65 V. In contrast, GaN-on-GaN HEMTs operated stably up to the analyzer’s limit of ±200 V, with current noise detected only at *V*_G_ = −170 V (Figure 6b). Joh et al. reported that the reliability of the GaN-based HEMTs under a high electric field can be greatly improved by reducing the stress on the epitaxial layer. Thus, the GaN-on-GaN HEMTs can withstand such a high electric field due to the homogeneous epitaxial structure relieving the stress on the epitaxial layer, as demonstrated by Raman spectroscopy [29]. In addition, the several results obtained for both samples had the same change rule: *I*_G_ ≈ *I*_D_ + *I*_S_. The representative results are shown in Figure 6a,b. Due to the voltage applied to the gate, it was reasonable that *I*_G_ was always larger than the other two currents for both samples. The difference between *I*_D_ and *I*_S_ of the GaN-on-SiC HEMTs was ~0.3 μA/mm before the critical voltage appeared, due to inferior epitaxial quality. On the contrary, due to the high material quality of the GaN-on-GaN structure, the *I*_D_ and *I*_S_ of the GaN-on-GaN HEMTs were almost exactly equal during the complete test.

At the end of each stress step, the Schottky and transfer characteristics of the device were detected to observe the degradation of the device’s performance. The voltage bias for the transfer characteristic was *V*_D_ = 10 V and *V*_G_ from −6 V to 2 V. The *I*_on/off_ of the device can be obtained from every transfer characteristic. The voltage bias for the Schottky characterization was *V*_D_ = vs. = 0 V and *V*_G_ from −10 V to 2 V. We defined the off-state gate current (*I*_G, off_) as the gate current at *V*_G_ = −10 V in the Schottky characteristic. The *I*_on/off_ and the *I*_G, off_ after each step were extracted from the transfer curve and Schottky curve, respectively. Figure 6c,d show the relationship between the *I*_on/off_ and *I*_G, off_/*I*_G, off (0)_ of the two devices as a function of reverse gate step stress, respectively. The higher material quality of the GaN-on-GaN structure reduced the current through the AlGaN barrier layer [30,31], so the *I*_G, off_ of the GaN-on-GaN HEMTs did not change significantly, even if the *V*_G_ exceeded the *V*_critical_. As a contrast, the *I*_on/off_ of GaN-based HEMTs on the SiC substrate decreased from 2.8 × 10^6^ to 1.3 × 10^4^, while the *I*_on/off_ of the GaN-on-GaN HEMTs varied from 1.7 × 10^7^ to 1.3 × 10^7^. The results proved that the homogeneous epitaxial structure had better reliability due to lattice matching.

**Figure 7:**
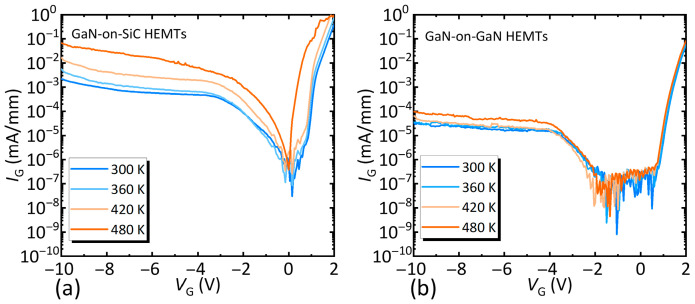
The variable-temperature Schottky characteristics of (**a**) the GaN-on-SiC HEMTs and (**b**) the GaN-on-GaN HEMTs after applying a −50 V gate stress.

We characterized the thermal stability of the two devices through variable-temperature Schottky characteristics. To ensure both devices were in the same state, the variable-temperature Schottky characteristics were characterized after applying a −50 V gate stress. The temperature increased from 300 K to 480 K in 60 K increments, as shown in Figure 7. The performance of the GaN-on-SiC HEMTs degraded sharply with increasing temperature after applied stress. In contrast, the GaN-on-GaN HEMTs demonstrated very weak temperature dependence in the Schottky characteristics after stressing. This result indicates that the GaN-on-GaN HEMTs maintain excellent thermal stability, even under prolonged electric field stress conditions.

**Figure 8:**
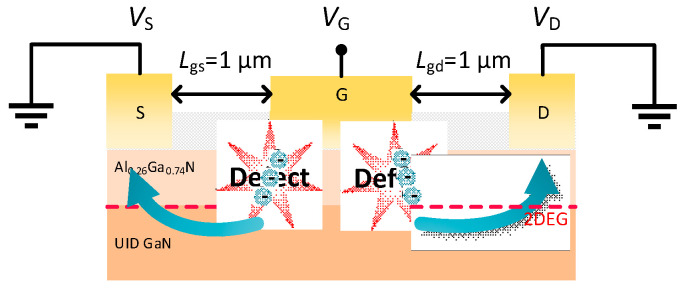
Schematic of leakage after the occurrence of inverse piezoelectric effect. The arrow indicates the direction of the leakage.

**Figure 9:**
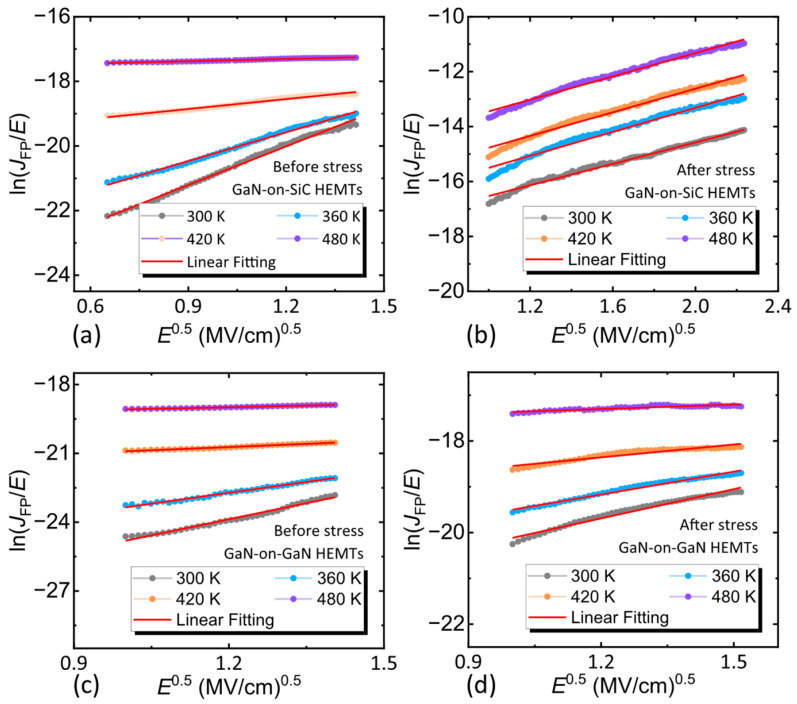
The FP emission fitting curves of the GaN-on-SiC HEMTs (**a**) before and (**b**) after stress. The FP emission fitting curves of the GaN-on- GaN HEMTs (**c**) before and (**d**) after stress.

It has been reported that the inverse piezoelectric effect of the device can produce defects at the edge of the gate [32], resulting in leakage, as shown in Figure 8. To investigate the gate leakage after the occurrence of inverse piezoelectric effect, variable-temperature (temperature was increased from 300 K to 480 K in 60 K steps) Schottky characterization was performed on free-state devices and devices that just reached *V*_critical_. The Frenkel–Poole (FP) emission mechanism related to dislocation (extracted from the inverse plateau region of the Schottky curve) and Fowler–Nordheim (FN) tunneling mechanism (extracted from the inverse linear region of the Schottky curve) were extracted [33,34]. Because the FN tunneling mechanism was mostly determined by characteristics such as the thickness of the AlGaN barrier layer, the FN tunneling fitting curve of the two devices did not differ significantly before and after stress [35], and the results of the FP emission fitting curves were shown in Figure 9. Obviously, after the inverse piezoelectric effect, the FP emission mechanism of the GaN-on-SiC HEMTs was enhanced, whereas the gate leakage current mechanism of the GaN-on-GaN HEMTs was almost unchanged. This proved that the GaN-on-GaN HEMTs did not introduce too many defect states after the inverse piezoelectric effect occurred, which also explained why the *I*_on/off_ and the *I*_G, off_ of the GaN-on-GaN HEMTs changed little after reaching the *V*_critical_. This result proved that the GaN-on-GaN HEMTs had better reliability than the GaN-on-SiC HEMTs, due to the superior quality of the epitaxial structure.

### 3.3. Off-State Drain Step Stress Characterization

**Figure 10:**
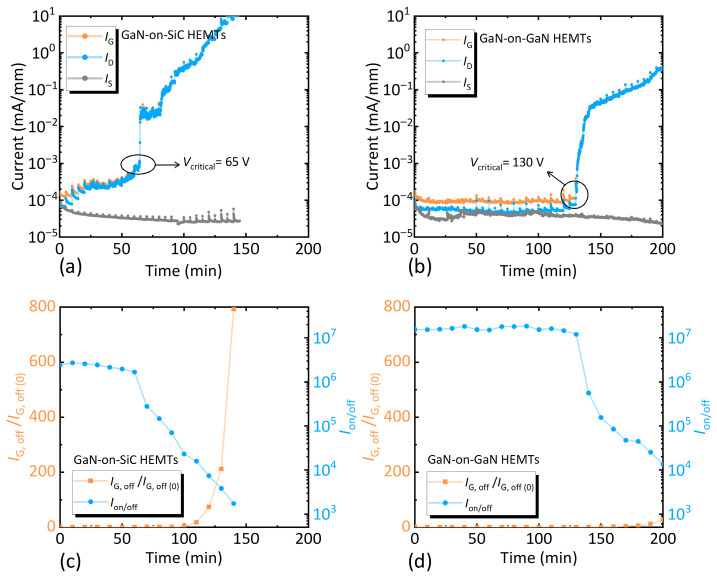
Changes in *I*_G_, *I*_S_, and *I*_D_ of (**a**) the GaN-on-SiC HEMTs and (**b**) the GaN-on-GaN HEMTs in off-state drain step stress experiments. Relationship between the *I*_on/off_ and *I*_G, off_/*I*_G, off (0)_ of (**c**) the GaN-on-SiC HEMTs and (**d**) the GaN-on-GaN HEMTs as a function of off-state step drain stress, respectively. *I*_G, off_/*I*_G, off (0)_ is defined as the ratio of the *I*_G, off_ after each step post-stress to the stress-free *I*_G, off_.

In addition, off-state drain step stress tests were conducted to assess reliability under high drain electric fields. For three-terminal devices, vs. was grounded, *V*_G_ = −10 V, and *V*_D_ was incrementally increased by 5 V every 5 min. The variation in three-terminal currents with drain stress was shown in Figure 10a,b. GaN-on-GaN HEMTs exhibited a *V*_critical_ nearly twice that of GaN-on-SiC HEMTs, with GaN-on-SiC devices failing at *V*_D_ = 150 V and GaN-on-GaN devices operating stably up to the analyzer’s maximum *V*_D_ of 200 V. As shown in Figure 10c,d, post-stress transfer and Schottky characteristics indicated that performance degradation accelerated beyond *V*_critical_ for both devices, but the GaN-on-SiC HEMTs suffered from more severe deterioration.

**Figure 11:**
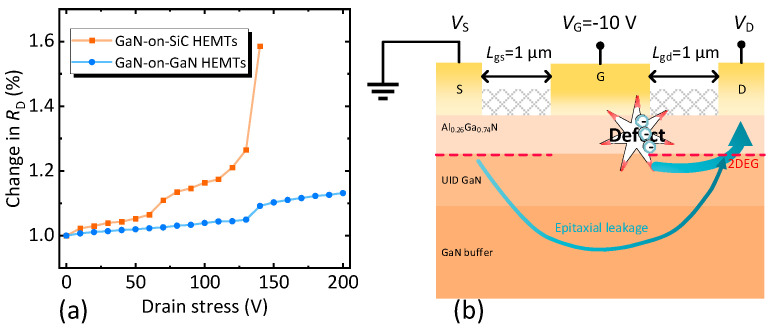
(**a**) The relationship between drain resistance (*R*_D_) and drain bias voltage of the two devices. (**b**) Schematic diagram of leakage of devices at a high field. The arrow indicates the direction of the leakage.

Analysis of leakage mechanisms in reverse gate step stress testing had already concluded that progressively intensifying stress introduced conductive defects into the AlGaN barrier layer. In the case of off-state drain step stress, further analysis of drain resistance (*R*_D_) revealed the origin of the device degradation. The relationship between *R*_D_ and drain bias voltage of the two devices was extracted, as shown in Figure 11a. In this work, *R*_D_ was measured by adopting the gate current injection method with *I*_Ginj_ = 10 mA/mm [36,37]. Obviously, the *R*_D_ of the two devices increased with the drain stress, which was consistent with past reports [29].

Combined with the conclusion of the reverse gate step stress, the degradation mechanism of the devices under a high field condition was obtained. When *V*_D_ < *V*_critical_, the trapping of AlGaN barrier layer in the active region increased with the electric field, resulting in *R*_D_ gradually increasing with the bias voltage [29]. Since the homogeneous epitaxial structure had a higher material quality, the *R*_D_ of the GaN-on-GaN HEMTs increased more slowly than that of the GaN-on-SiC HEMTs. The main leakage channel at this time was the impurities in the buffer and the original existing defects [30], as shown in Figure 11b. When *V*_D_ > *V*_critical_, lattice defects were introduced into the AlGaN barrier layer due to the inverse piezoelectric effect [38]. Emerging defects not only enhanced the trapping but also formed conductive channels [30]. As illustrated in Figure 11b, the main leakage channel at this time occurred in the AlGaN barrier layer between the gate and the drain, resulting in a simultaneous increase in *I*_G_ and *I*_D_ for both devices in the off-state drain step stress test. At the same time, the *R*_D_ tended to increase sharply with increasing drain stress, due to electrons trapped by the defects formed in the AlGaN barrier layer [39]. As a result, the *I*_on/off_ of the two devices decreased rapidly and the performance of the off-state (e.g., *I*_G, off_) deteriorated sharply. In the whole process, because the GaN-on-GaN HEMTs were lattice-matched, they had a higher *V*_critical_, beyond which the device did not immediately fail. The lattice-matched structure delayed this degradation, confirming their robust epitaxial quality.

### 3.4. On-State Electrical Stress Characterization

**Figure 12:**
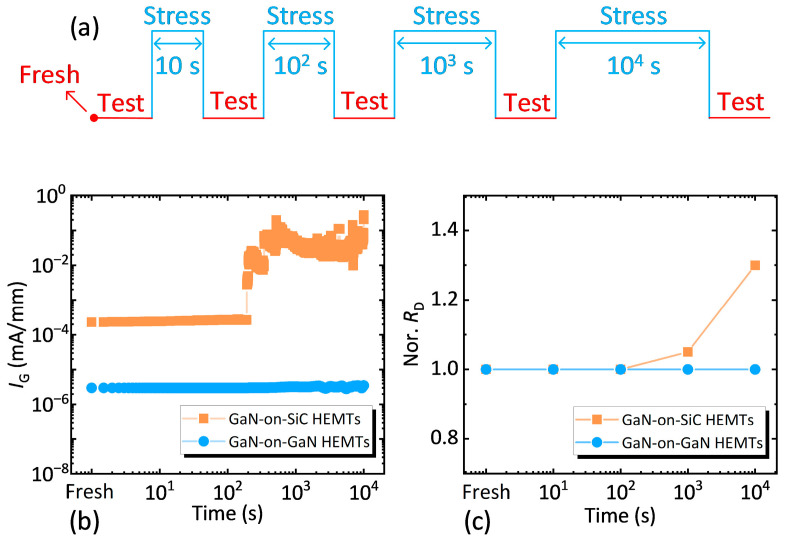
(**a**) Schematic of the on-state stress test setup for both samples. Relationship between (**b**) *I*_G_ and (**c**) normalized *R*_D_ with on-state stress time for both samples. Normalized *R*_D_ is defined as the ratio of the *R*_D_ after each step-stress to the stress-free *R*_D_.

Furthermore, on-state electrical stress tests were performed to simulate real-world operating conditions, where devices are exposed to both high voltage and current. Stress was applied at *V*_D_ = 30 V and *V*_G_ = 0 V for durations of 10 s, 100 s, 1000 s, and 10,000 s, respectively. Static electrical characteristics and *R*_D_ were evaluated pre- and post-stress. The schematic diagram of the on-state stress reliability experiment for the device is illustrated in Figure 12a.

During on-state operation, dislocations promote trap formation and electron capture [40], with high temperatures (from *V*_D_ × *I*_D_ power dissipation) and bias stress accelerating defect generation [39,41]. As shown in Figure 12b,c, GaN-on-SiC HEMTs exhibited rapid degradation of *I*_G_ and *R*_D_ after 1000 s of stress, whereas GaN-on-GaN HEMTs maintained a stable performance throughout the 10,000 s test. This stability is attributed to the low dislocation density of the GaN-on-GaN epitaxial structure, which minimizes trap formation and electron trapping—critical for long-term reliable operation in power-switching applications.

## 4. Conclusions

This study systematically investigates the influence of dislocation density on the reliability of GaN HEMTs under high electric fields, comparing GaN-on-GaN and GaN-on-SiC devices through comprehensive electrical and stress tests. GaN-on-GaN HEMTs achieve a record BFOM of 950 MW/cm^2^, enabled by a high BV of 755 V and a low *R*_ON-SP_ of 0.6 mΩ·cm^2^. The homoepitaxial structure reduces dislocation density by 83.3% and minimizes initial tensile stress, endowing the devices with exceptional reliability under reverse gate step stress, off-state drain step stress, and on-state electrical stress. These results validate that reducing dislocation density is a key strategy to enhance the performance and reliability of GaN HEMTs, positioning the GaN-on-GaN technology as a transformative solution for advanced power electronics. With its unique combination of high efficiency and robustness, GaN-on-GaN HEMTs hold great promise for demanding applications such as electric vehicles, renewable energy systems, and industrial power converters.

## Data Availability

The data presented in this study are available on request from the corresponding author.
